# Classification based on extensions of LS-PLS using logistic regression: application to clinical and multiple genomic data

**DOI:** 10.1186/s12859-018-2311-2

**Published:** 2018-09-06

**Authors:** Caroline Bazzoli, Sophie Lambert-Lacroix

**Affiliations:** 1 Laboratoire Jean Kuntzman, Univ. Grenoble-Alpes, 700 avenue centrale, Saint Martin d’Hères, 38401 France; 2 TIMC-IMAG, Univ. Grenoble-Alpes, 5 Avenue du Grand Sablon, La Tronche, 38700 France

**Keywords:** Classification, Clinico-genomic model, High-dimensional data, Logistic regression, LS-PLS

## Abstract

**Background:**

To address high-dimensional genomic data, most of the proposed prediction methods make use of genomic data alone without considering clinical data, which are often available and known to have predictive value. Recent studies suggest that combining clinical and genomic information may improve predictions. We consider here methods for classification purposes that simultaneously use both types of variables but apply dimensionality reduction only to the high-dimensional genomic ones.

**Results:**

Using partial least squares (PLS), we propose some one-step approaches based on three extensions of the least squares (LS)-PLS method for logistic regression. A comparison of their prediction performances via a simulation and on real data sets from cancer studies is conducted.

**Conclusion:**

In general, those methods using only clinical data or only genomic data perform poorly. The advantage of using LS-PLS methods for classification and their performances are shown and then used to analyze clinical and genomic data. The corresponding prediction results are encouraging and stable regardless of the data set and/or number of selected features. These extensions have been implemented in the R package lsplsGlm to enhance their use.

**Electronic supplementary material:**

The online version of this article (10.1186/s12859-018-2311-2) contains supplementary material, which is available to authorized users.

## Background

Over the past 15 years, progress in the generation of high-dimensional genomic data has raised high expectations in biomedical research. Large-scale technologies have produced a wide variety of genomic features, such as mRNA-gene expression, DNA methylation, microRNA, and copy number alterations (CNAs), among others. Many genomic data of these types have been generated and analyzed in numerous studies with the aim of predicting a specific outcome [1, 2]. In this article, we focus on binary class prediction where the outcome can be for instance alive/dead, or therapeutic success/failure. Most of these studies [3–7] include clinical data in addition to genomic data, using most of the proposed prediction methods with only genomic data, which involves some statistical issues. In genomic studies, the number of samples *n* is often relatively small compared to the number of covariates *p*, and collinearity between measurements occurs. Unless a preliminary step of variable selection is performed, the standard classification methods are not appropriate. To address this “large *p* small *n*” problem, variable selection or dimensionality reduction methods or a combination of both can be used. We focus here only on those dimensionality reduction methods that aim at summarizing the numerous predictors in the form of a small number of new components (often linear combinations of the original predictors). The traditional approach is principal component regression (PCR)[8], an application of principal component analysis (PCA) to the regression model. PCA is applied without considering the link between the outcome and the independent variables. An alternative method is the partial least square (PLS) method [9], which takes this link into account.

In recent studies [10–12], most complex diseases have been shown to be caused by the combined effects of many diverse factors, including genomic and clinical variables. This has led to an emerging research area of integrative studies of clinical and genomic data, which we will refer to as clinico-genomic models. Some strategies to combine these two kinds of data have been reviewed in a paper written by [13] to adress predictive clinico-genomic models. More extensive overviews are available in [14], where advantages and disadvantages are given for each strategy. Regarding the dimensionality reduction strategy, one possible way to handle the high dimensionality of genomic data is to first apply dimensionality reduction techniques to only the genomic data set. In the second step, the selected genomic variables are merged with the clinical variables to build a classification model on the combined data set. We refer to this as a two-step approach. Most previous techniques select the topmost discriminative genomic features and then combine those features into a combined score for future model development. In the same way, [15] suggest an approach combining PLS dimensionality reduction with a prevalidation technique and random forests, applied with both the new components and the clinical variables as predictors. These papers mainly describe methods using PLS dimensionality reduction to treat high-dimensional data. Even if any type of dimensionality reduction method can be incorporated, these two-step approaches cannot account for the relationship existing between two data sets. Indeed, this reduction is achieved without considering into account the link between the response variable and the clinical data.

An alternative approach could be to use an iterative procedure well suited to extracting relevant information from the genomic data in combination with clinical variables. One idea is to use the principle of backfitting procedures developed in the context of multidimensional regression problems and derived for generalized additive models [16], estimating additive components successively in a nonparametric manner. Specifically, this involves repeatedly fitting nonparametric regression of some partial residuals on each covariate. For each regression, a new additive component is estimated, which in turn yields new partial residuals; this process is iterated until convergence. Then, updates based on relevant information from both data types takes place within the iterations. This one step approach was developed by [17] in the regression Gaussian context for chemometrics. Nonparametric regression is replaced with PLS regression for the data to be compressed and ordinary least squares (OLS) regression for other data, so-called LS-PLS. The PLS scores are thus incorporated into the OLS equations in an iterative fashion to obtain a model for both the clinical variables and the genomic ones. The authors conclude that the method seems to involve more information from the experiment and return lower variance in the parameter estimates.

The purpose of this paper is thus to adapt this one-step LS-PLS procedure to logistic regression models. To carry this out, we first need to extend PLS in this context. Some studies proposing an adaptation of PLS for classification problems have been published [18–20]. In this paper, we focus on adapting these extensions to LS-PLS to address the logistic regression model. The method section gives the details of the original LS-PLS approach corresponding to Gaussian linear regression, that corresponding to linear logistic regression and three novel extensions of LS-PLS for logistic regression models. The simulation study conducted to evaluate these approaches, and a demonstration on two real data sets containing both clinical information and multiple genomic data types (gene expression and CNA) are presented in the results section.

## Results

### Simulation study

The aim of the simulation study is to compare the different prediction methods developed based on clinical and/or gene expression variables. We simulated data sets with a range of predictor collinearity and with different functional relationships between the response, *Y*_*i*_, and the predictors **X**_*i*·_ and **D**_*i*·_ to mimic gene expression and clinical variable data. For an individual *i*=1,...,*n*, with *n*=100, we simulated $ {Y}_{i} \sim \mathcal {B}({\pi _{i}})$ with $\pi _{i}=\left [1\; {\mathbf {D}}_{i\cdot }^{T} \;{\mathbf {X}}_{i\cdot }^{T}\right ]{\boldsymbol {\gamma }}$, where ***γ***, the vector of regression parameters, defined as ***γ***=[*γ*_1_ ***γ*D***T* ***γ*X***T*]^*T*^. We fixed *γ*_1_=−2.5, ***γ***_**D**_={{0.5}^4^} and ***γ***_**X**_={{0}^475^,{0}^475^,{0.1}^25^,{0.1}^25^}. The matrix **X** of size *n*×*p* (with *p*=1000) has been simulated as **X**=(**X**^1^,**X**^2^,**X**^3^,**X**^4^), where ${\mathbf {X}}^{k} \sim N\left (0_{bs^{(k)}},{\boldsymbol {\Sigma }}_{{\mathbf {X}}}^{k}\right)$ with {***Σ*X***k*}_*ij*_=*c*_*k*_*ρ*^|*i*−*j*|^, *k*=1,...,4, *i*,*j*=1,...,*bs*^(*k*)^, where *c*_1_=8, *c*_2_=4, *c*_3_=2, and *c*_4_=1, *bs*^(1)^=*bs*^(2)^=475, *bs*^(3)^=*bs*^(4)^=25, and *ρ*=0.9. Regarding the matrix **D** of size *n* x *q* (with *q*=4), we used *N*(0_*q*_,***Σ***_**D**_) with {***Σ***_**D**_}_*ij*_=*ρ*^|*i*−*j*|^, with *i*, *j*=1,...,*q* and *ρ*=0.5. According to this model, we generated 100 training sets of size *n*=100 and 100 test sets of size 450. Note that the context of this simulation is unfavorable for LS-PCR. Indeed, since the variable blocks that are not active in the model possess the strongest variability, they stand out from among the first *κ* components of the PCA.

Our proposed extensions, i.e., LS-PLS-IRLS, IR-LS-PLS, and R-LS-PLS, are then applied to the simulated data sets. To compare the accuracy and efficiency of the latter, the GLM is applied to clinical data alone, and R-PLS is applied to gene expression data alone. The usual method based on PCR is also considered. In our context, gene expression data are replaced with the first *κ* principal components of **X** (obtained by PCA), which constitute the directions of maximal variability in the data of **X**, without considering the response variable **Y**. Let **T** be the matrix of columns, that correspond to the first *κ* PCA scores associated with **X**. The parameters are then estimated by running IRLS (**Y**,[**D** **T**]). This approach is called least squares principal component regression (LS-PCR). For all approaches, the optimal number of PLS or PCR components is selected by choosing *κ* values in the range of 1,...,*κ*_*max*_, with *κ*_*max*_=1, 4 and 8, by a fivefold cross-validation on each of the 100 training sets. That is, each training set is split fivefold into a test set, containing one-fifth of the data, and a learning set, containing the remaining four-fifths of the data. We retain the value of *κ*, which minimizes the misclassification rate over this fivefold cross-validation. This is also employed for R-LS-PLS, where the *κ* value and *λ* for 6 *log*_10_−linearly spaced points in the range [10^−3^;100] are simultaneously determined by this cross-validation method.

As referenced in [15], although variable selection is not always necessary as a preliminary step to PLS-based classification, some authors argue that accuracy is improved in the high-dimensional setting, especially when indeed few relevant variables exist. Many variable selection procedures are available in the literature. In the present article, sure independence screening (SIS) [21] is performed to select relevant gene expression variables *p*_*red*_=500 such that *p*_*red*_ is strictly smaller than *p*. The SIS procedure involves ranking features according to marginal utility, namely, each feature is used independently as a predictor to determine its usefulness for predicting the response. Specifically, the SIS procedure ranks the importance of features according to their magnitude of marginal regression coefficients.

To evaluate prediction performance, mean misclassification rates and the area under the curve (AUC) are computed on the 100 test sets for each method. The rates of convergence are also assessed for LS-PCR and those methods based on the PLS algorithm. Simulations and analyses are performed using the R software, version 3.1.2.

**Fig. 1:**
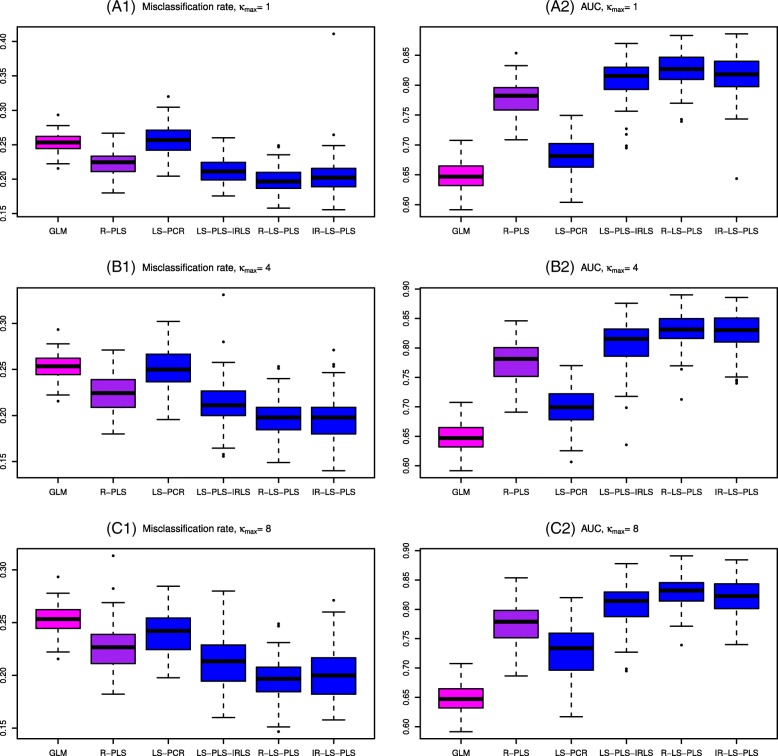
Boxplot of the misclassification rates (left part) and AUCs (right part) from the 100 simulated data sets. The results were obtained using the six methods and according to different *κ*_*max*_: (A1, A2): *κ*_*max*_=1; (B1, B2): *κ*_*max*_=4; (C1, C2): *κ*_*max*_=8. GLM and R-PLS denote the misclassification rates and AUCs obtained from applying the GLM to the clinical data alone and PLS to gene expression data alone, respectively. LS-PCR denotes the approach derived from PCR, where gene expression data are analyzed using PCA and IRLS can thus be applied to the merged data set of PCA scores and clinical data. LS-PLS-IRLS, R-LS-PLS, and IR-LS-PLS denote the misclassification rates and AUCs obtained from the newly proposed LS-PLS approaches combining expression and clinical data. For clarity, we use a color code to indicate the predictions: pink when from clinical data alone, purple when from expression gene data alone and blue for the results of methods combining both types of variables. The number of gene expression variables to pre-select *p*_*red*_ is set to 500 in the SIS procedure

The simulation results are summarized in Fig. 1 and Table 1, which were produced based on the 100 simulated data sets. They depict the distributions of misclassification rates, AUCs and convergence rates in percent. For this simulation study, the two classes are much less distinguishable by the clinical data than by the gene expression data, which is confirmed in Fig. 1. Analyses of the clinical features alone by the GLM and genomic data alone using R-PLS are less informative in predicting the outcome than those of the approaches combining both types of variables. All approaches integrating clinical and genomic data, except LS-PCR, show comparable discrimination rates. The method using PCR increases the misclassification rates and decreases the AUC as *κ*_*max*_ decreases. Quite surprisingly, even with *κ*_*max*_=4 or 8, LS-PCR does not achieve the performance of the LS-PLS approaches. According to the model structure, we would expect LS-PCR to identify the two active components and thus to yield similar results. For each case of *κ*_*max*_, R-LS-PLS seems to be better than the two other extensions of PLS (LS-PLS-IRLS and IR-LS-PLS), even though the median misclassification rates of R-LS-PLS and IR-LS-PLS are very similar to each other. The analysis of the variance of misclassification error rates follows the same trend as previously described, i.e., the misclassification error rate. R-LS-PLS leads to less variability than the other methods. The same behavior is also observed in the resulting convergence rates reported in Table 1. R-LS-PLS does not show convergence problems (all rates equal 100%). The convergence rate of LS-PLS-IRLS is much lower than that of R-LS-PLS, probably due to numerical instability of the methods when *n* is smaller than the number of variables. Notably, the interpretation of the convergence rate of IR-LS-PLS is seriously limited by the lack of an optimum criterion in the approach. One explanation could be that when solving the weighted LS problem in each IRLS iteration with LS-PLS, the global problem cannot be rewritten as the optimization of a loss function.

**Table 1:** Rates of convergence (%) from the 100 simulated data sets for the five methods, according to different *κ*_*max*_:1,4 and 8

*κ*_*max*_	R-PLS	LS-PCR	LS-PLS-IRLS	R-LS-PLS	IR-LS-PLS
1	100	100	71	100	22
4	100	100	41	100	76
8	100	99	44	100	78

R-PLS denotes the results from the analysis of gene expression alone. LS-PCR denotes the approach derived from PCR, where gene expression data are analyzed using PCA and IRLS can thus be applied to the merged data set of PCA scores and clinical data. LS-PLS-IRLS, R-LS-PLS, and IR-LS-PLS denote the rates of convergence from the newly proposed approaches combining expression and clinical data. The number of gene expression variables to preselect *p*_*red*_ is set to 500 in the SIS procedure

Note that the noninfluential variables having the highest variances may seem unrealistic since the influential gene expression variables can have higher variances than the noninfluential ones in practice. To make the simulation results more robust with respect to a potential bias towards an overoptimistic performance of our approaches, we have chosen to attribute a stronger variability to the noninfluential variables. We have thus reconsidered the same simulation example but inverted the variances levels. Surprisingly, we obtain similar results; the LS-PCR method leads to poorer performance even if *κ*_*max*_ is equal to 8 (see Additional file 1).

### Application to real data sets

We apply the extensions presented previously to two publicly available real data sets for which both clinical and genomic variables are available. Similar to the simulation study, to validate procedures of the clinico-genomic models, we compare the combined clinico-genomic model accuracy and AUC with those of the models built either with genomic data or clinical data alone. We apply and compare all the methods considered in the simulation study. On both real data sets, we perform a re-randomization study on 100 random subdivisions of the data set into a learning set and a test set. For the first one, we choose a test set size equal to one-third the data (2:1 scheme of [22]); considering the size of the second data set, a ratio of 30 (learning set) to 70 (test set) has been used. The SIS procedure is applied to genomic data, as in the simulation study, considering different numbers of selected genes *p*_*red*_: 50, 100, 500 and 750. For the real data, the *κ* range is {1,2,...,5} and the *λ* range is given by 6 log10-linearly spaced points in the range [10^−3^;100].

#### Gene expression : central nervous system data

The first data set was obtained from [23], which has been used to predict the response of childhood malignant embryonal tumors of the central nervous system (CNS) to therapy. The data set is composed of 60 patient samples, with 21 patients having died and 39 having survived within a period of 24 months; gene expression data and clinical data are available for each patient. There are 7129 genes, and the clinical features are sex (binary), age (ordinal), chemo CX (binary) and chemo VP (binary). The original data set contains the clinical variable change stage, which has been omitted due to its large number of categories.

**Fig. 2:**
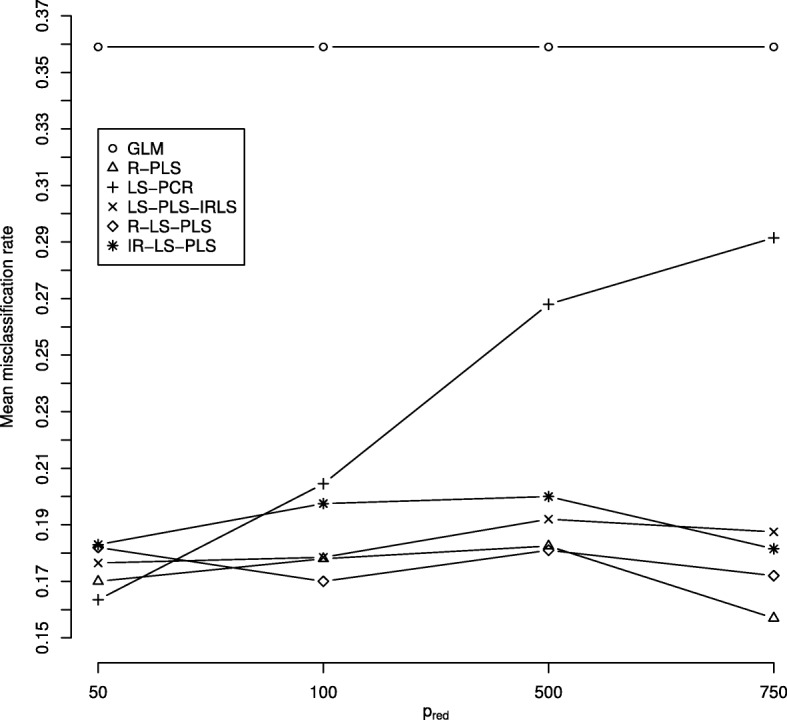
Mean misclassification rates from the central nervous system (CNS) data set using the six methods considering different numbers of selected genes *p*_*red*_: 50, 100, 500 and 750. GLM and R-PLS denote the misclassification rates and AUCs obtained from applying the GLM to the clinical data alone and PLS to the gene expression data alone, respectively. LS-PCR denotes the approach derived from PCR, where gene expression data are analyzed using PCA and IRLS can thus be applied to the merged data set of PCA scores and clinical data. LS-PLS-IRLS, R-LS-PLS, and IR-LS-PLS denote the misclassification rates obtained from the newly proposed LS-PLS approaches combining expression and clinical data. For each method, a line is drawn to connect symbols to improve readability

**Fig. 3:**
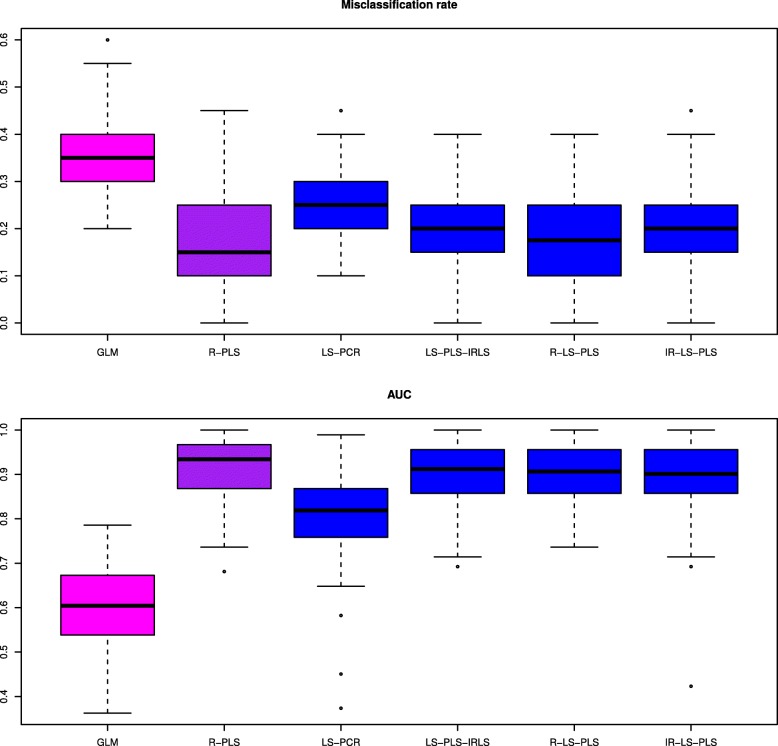
Distribution of misclassification rates and AUCs for central nervous system data, estimated from 100 samples using the six methods. GLM and R-PLS denote the misclassification rates and AUCs obtained from applying the GLM to the clinical data alone and PLS to the gene expression data alone, respectively. LS-PCR denotes the approach derived from PCR, where gene expression data are analyzed using PCA and IRLS can thus be applied to the merged data set of PCA scores and clinical data. LS-PLS-IRLS, R-LS-PLS, and IR-LS-PLS denote the misclassification rates and AUCs obtained from the newly proposed LS-PLS approaches combining expression and clinical data from the central nervous system data set. The number of gene expression variables to pre-select *p*_*red*_ is set to 500 in the SIS procedure. The color code for the methods is similar to that in Fig. 1

Figure 2 depicts the mean misclassification rates according to the number of selected genes *p*_*red*_ obtained for the analysis of the CNS data. This data set presents a situation in which, gene expression data alone (R-PLS) performed better than clinical data alone (GLM), with the lowest misclassification rates regardless of the value of *p*_*red*_ (0.35 for GLM and approximatively 0.17 for R-PLS). Except for LS-PCR, the proposed procedures integrating clinical and genetic features perform well with corresponding misclassification rates ranging from 0.16 to 0.20. These findings are not influenced by the number of significant gene expression variables. However, the misclassification rate from LS-PCR increases as *p*_*red*_ grows. We consider that the information necessary to correctly predict the response could be concentrated in only a set of 50 genes. As provided, overall, the prediction performances of R-PLS are close to those achieved using the newly proposed LS-PLS approaches. The accuracy of the prediction approaches for the CNS using only 500 selected genes is detailed in Fig. 3. As already noted, the performance in relation to the clinical data when predicting the response is poor. The R-LS-PLS method attains the highest median accuracy, close to the median misclassification rate achieved when analyzing only gene expression data via PLS (R-PLS). The prediction results of LS-PLS-IRLS and IR-LS-PLS are very similar and better than those of R-LS-PLS. We note the large variability of the misclassification rates for all proposed LS-PLS methods. For this study, the worst predictions are obtained using the LS-PCR method, indicating the poor performance of PCR in treating information stored in high-dimensional data. Plots similar to those in Fig. 3, corresponding to the three other values of *p*_*red*_ are given in Additional file 2.

#### Copy number alterations: breast cancer data

The second original data set [10, 24] contains information on 2173 primary breast tumors, integrating somatic CNAs and long-term clinical follow-up data. Different types of data are merged based on the sample IDs. The data of a total of 1349 primary breast tumors (684 from patients with ER-positive (ER+) status and 221 with ER-negative (ER-) status) are given, including the clinical variables (grade (nominal), tumor stage (ordinal), human epidermal growth factor receptor 2 (HER2) status (binary), tumor size (numeric), progesterone receptor status (binary)) and CNA measurements. The goal here is to predict the ER stratification of a novel breast tumor to select the appropriate treatment for breast cancer. Concerning somatic CNAs, the data set used in this paper is prepared as described in the original manuscript [24], yielding 22544 somatic mutations. The data were downloaded from the TCGA data portal (https://tcga-data.nci.nih.gov/).

**Fig. 4:**
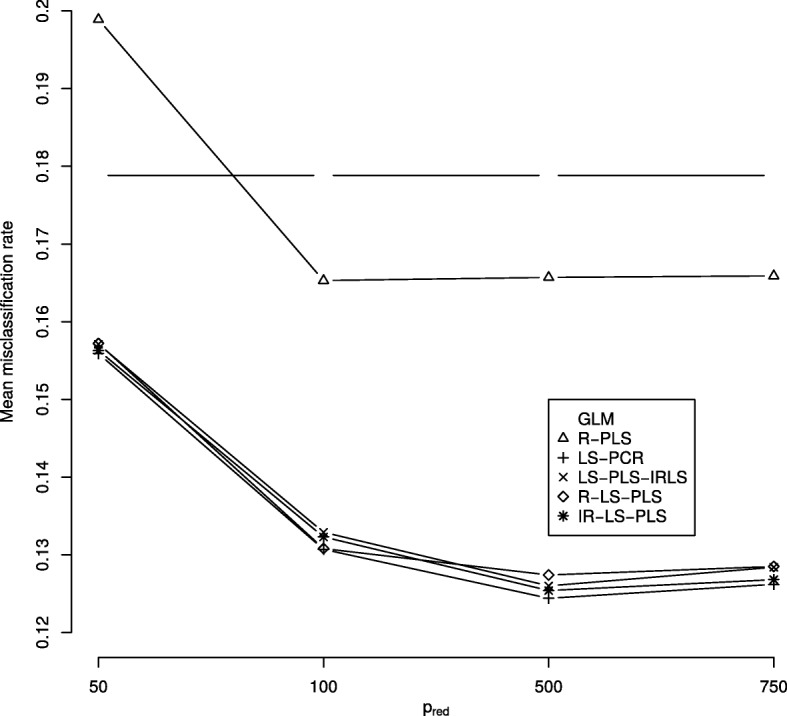
Mean misclassification rates from the somatic CNA data set using the six methods considering different numbers of selected genes *p*_*red*_: 50, 100, 500 and 750. GLM and R-PLS denote the misclassification rates obtained from applying the GLM to the clinical data alone and PLS to the CNA data alone, respectively. LS-PCR denotes the approach derived from PCR, where CNA data are analyzed using PCA and IRLS can thus be applied to the merged data set of PCA scores and clinical data. LS-PLS-IRLS, R-LS-PLS, and IR-LS-PLS denote the misclassification rates obtained from the newly proposed LS-PLS approaches combining CNA and clinical data. For each method, a line is drawn to connect symbols to improve readability

**Fig. 5:**
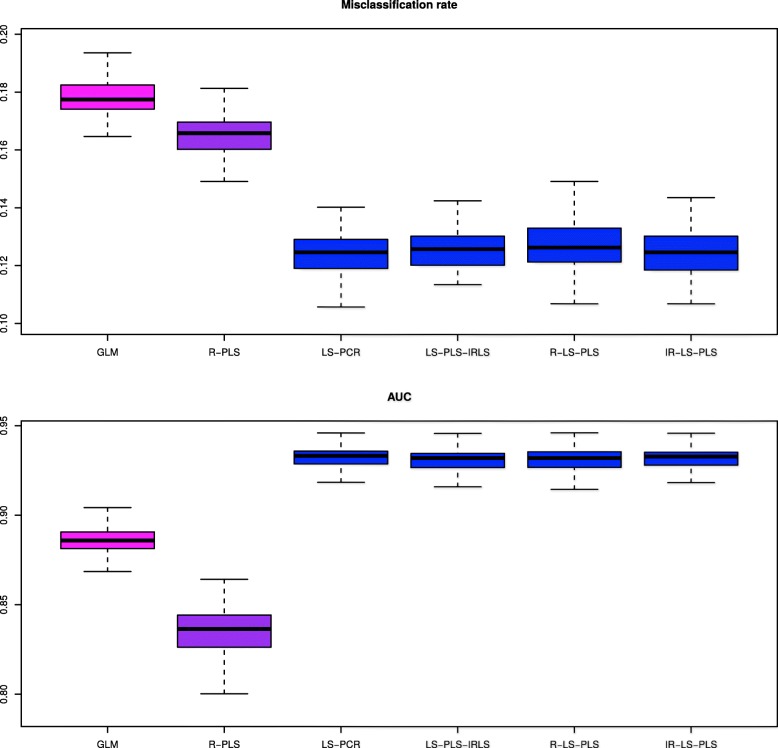
Distribution of misclassification rates and AUCs for the somatic CNA data estimated based on 100 samples using the six methods. GLM and R-PLS denote the misclassification rates and AUCs obtained from applying the GLM to the clinical data alone and PLS to the CNA data alone, respectively. LS-PCR denotes the approach derived from PCR, where CNA data are analyzed using PCA and IRLS can thus be applied to the merged data set of PCA scores and clinical data. LS-PLS-IRLS, R-LS-PLS, and IR-LS-PLS denote the misclassification rates and AUCs obtained from the newly proposed LS-PLS approaches combining CNA and clinical data from the brest cancer data set. The number of gene expression variables to pre-select *p*_*red*_ is set to 500 in the SIS procedure. The color code for the methods is similar to that used in Fig. 1

We report in Fig. 4, the mean misclassification rates obtained for the most pertinent covariates from the SIS procedure *p*_*red*_ for all methods. Here, we have the case where the use of clinical data alone or genomic data alone does not offer good predictors of ER stratification. Indeed, we observe a major gain in misclassification rates when the response variable is predicted using either the LS-PLS or LS-PCR approaches regardless of the value of *p*_*red*_. More specifically, the rates decrease to values between *p*_*red*_=50 and *p*_*red*_=500 and no longer change. The optimal misclassification rate is close to 0.13 with *p*_*red*_=500. Figure 5 shows a boxplot of the misclassification rates and the AUCs for *p*_*red*_=500. The analysis of the CNA data improves only the prediction accuracy yielded by the clinical variables alone. The median misclassification rate obtained using R-PLS is smaller than that obtained via the GLM. The four methods combining clinical and genomic data provide similar and significantly better misclassification rates and AUCs compared to those of both the GLM and R-PLS. These findings suggest that CNA data perform slightly better than clinical data, though the integration of both features is more effective in predicting the response. Plots similar to those in Fig. 5, corresponding to the three other values of *p*_*red*_, are given in Additional file 3.

## Discussion

The three extensions of the LS-PLS and PCR-type approaches have been implemented in the R package lsplsGlm. A clinico-genomic model that can predict a binary outcome using dimensionality reduction methods would be a useful computing tool for integrating clinical and gene expression data. In general, the methods using only clinical data or only genomic data perform less well.

We show that it is not always advisable to use the PCR-type method, which can lead to suboptimal results that depend on the data type and the number of selected features and therefore the relation between the response variable and the covariate structure. Indeed, in PCR, the principal components that are dropped correspond to the near-collinearities among the genetic data. PCR does not consider the response variable when determining which principal components to drop. Although cross-validation has been used to select the optimal number of components, this decision is based mainly on the magnitude of the variance of the components since in PCA, the dependence on the response variable is weak when compared to PLS. The LS-PLS extensions have been shown to be capable of simultaneously analyzing both clinical and genetic data. We also demonstrated that the LS-PLS methods have several advantages over other approaches. The corresponding prediction results are quite accurate and stable regardless of the data set and/or the number of selected features, which is not the case for LS-PCR. Concerning the comparison among the three LS-PLS extensions, we first mention the convergence problems for the LS-PLS-IRLS and IR-LS-PLS methods. We note that for the LS-PLS-IRLS method, the convergence problem can be linked to the GLM algorithm, whereas for the R-LS-PLS method, it is related to the algorithm itself.

In practice, dependencies frequently occur between clinical and gene expression data, which is why the question of the additional predictive value of gene expression data to clinical data plays an important role in the literature [12, 25]. When clinical or gene expression covariates are considered separately, well-performing prediction rules can be achieved, but additional value can be obtained by considering the gene expression when the clinical covariates are still present in the model. Therefore, it seems interesting to consider settings in which correlations exist between the clinical data and the gene expression data. From a conceptual point of view, the three methods have the same approach regarding the issue of collinearity present among clinical and genomic data. Indeed, for the three approaches, the matrix of gene expressions is orthogonalized on that of the clinical data, which is not the case in the PCR approach. In Additional file 4, we consider examples with **D** and **X** to be generated such that some of the variables among these two data sets are correlated. We have varied these correlations and studied the behaviors of the different methods. We observe that R-LS-PLS always does better regardless of the collinearity level. The other two extensions of LS-PLS are much more variable and are less satisfactory on average, although they tend to improve as the collinearity level increases. We believe that this outcome is due to the convergence problem of these two LS-PLS extensions.

Regarding the comparison with the two-step approaches, the results obtained from the LS-PLS approaches presented here are different from the findings of [15], where data are analyzed using a two-step approach based on random forests (RF) and PLS reduction. Our approaches were applied to the breast cancer gene expression data (results not shown here) considered in [15]. In this study, the best rate of misclassification was 0.2269 on average, while the worst was 0.2981. In the study in [15], regarding methods based on PLS, the best rate of misclassification was 0.30 on average, while the worst was 0.43. Hence, the one-step approach using the two data sets simultaneously seems better than the two-step approach using the two data sets separately.

A study by [26] on an extension of Integrative mixture of experts (ME) models for combining clinical and gene markers to improve cancer prognosis has been published. They illustrate the performance of the methodology on three cancer studies and, particularly, on CNS data sets. Even if the study using integrative ME cannot be considered as a dimensionality reduction approach, the authors first assess the classification performance on each separate data set, as in our study. Then, they compare the integrative ME with the logistic regression and PLS-RF of [15] on the combined data sets. Using three different preselection variable steps, an evaluation in which was varied *p*_*red*_ between 5 and 30 was performed. They show the important role of the gene selection step in the predictive ability of these models. Compared with our findings, regardless of the variable selection step, the average error rates obtained using the integrative ME approach are higher than those obtained using the extensions of LP-PLS for logistic regression with *p*_*red*_=50. When the data sets are combined and with 30 genes preselected, the average classification error rates obtained via the integrative ME approaches are greater than 30%, while they are less than 20% for LP-PLS extensions.

Determining the appropriate number of genomic features in the first step is difficult. The number of features may impact the comparison between the additive performances corresponding to clinical and genomic variables. For example, if too many features are selected from genomic data, the clinico-genomic model may be overfit in the second phase. On the other hand, if too few genomic factors are retained, then the predictive capability of the genomic factor can be underestimated. We may conclude that the model’s performance was not improved by the addition of large numbers of genes but was improved by the interplay of significant clinical features and genomic profiles.

This work constitutes a first step towards the extension of LS-PLS. In the present study, we consider only the case of LS-PLS for classification problems. Due to the large number of studies modeling survival using gene expression [27, 28], another natural extension of this work is to use LS-PLS approach to generate survival prediction models. The outcome would be a right-censored time-to-event such as the time to death or the time to next relapse, and Cox regression models must be considered.

Recently, some sparse versions of PLS have been proposed for high-dimensional classification problems in genome biology [29–31]. They aim to achieve variable selection and dimensionality reduction simultaneously for one type of data and they show that the combination of both increases the prediction performance and selection accuracy. This suggests that a subsequent extension of PLS could be carried out to achieve a “sparse” version of LS-PLS in the challenging task of combining both clinical and genomic factors.

## Conclusion

Despite the great potential of clinico-genomic integration, the topic is still in its elaboration phase. In general, integrating heterogeneous data sets such as clinical and genomic data is an important issue. We have proposed three extensions of LS-PLS approaches for logistic regression models to analyze both clinical and genomic data. The advantage of using those methods for classification and their performances are shown and then used to analyze clinical and genomic data. The corresponding prediction results are encouraging and stable regardless of the data set and/or number of selected features. These extensions have been implemented in the R package lsplsGlm to enhance their use.

## Methods

### Original LS-PLS approach

In the following, we consider situations where we have both partly collinear measurements, such as high-dimensional genomic data, and orthogonal (or near-orthogonal) design variables on one side that we want to relate to a response value on the other side. We denote the design matrix associated with the collinear measurements as **X**. For instances, in genomic samples, expression levels of the *p* genes for the *n* genomic samples are collected in this *n*×*p* data matrix **X**. The clinical variables are stored in matrix **D** of size *n*×*q*.

The combination of least squares (LS) and PLS (called LS-PLS) was first introduced in the Gaussian context by [17]. LS-PLS involves an iterative procedure: the first step is to use OLS on $\tilde {{\mathbf {D}}}$ to predict **Y** and compute the residuals. The matrix $\tilde {{\mathbf {D}}}$ is defined as $\tilde {{\mathbf {D}}} = \left [1_{n} \; {\mathbf {D}}\right ]$, with 1_*n*_=(1,⋯,1)^*T*^. Then, PLS is performed between **X** and the residuals to obtain the matrix of PLS scores **T** (of size *n*×*κ*). **T** is combined with $\tilde {{\mathbf {D}}}$ in a new OLS regression to predict **Y**. New estimates for the residuals of **Y** on $\tilde {{\mathbf {D}}}$ are obtained, keeping only the residuals associated with $\tilde {{\mathbf {D}}}$ in the OLS of *Y* on $[\tilde {{\mathbf {D}}}, {\mathbf {T}}]$. This algorithm is repeated until convergence. The authors suggest orthogonalizing **X** on $\tilde {{\mathbf {D}}}$. The orthogonalized variant is better suited for situations where the focus is on identifying the unique information in each matrix. The matrix **X** is thus projected into an orthogonal space spanned by the design variables of $\tilde {{\mathbf {D}}}$:

$${\mathbf{X}}_{Orth}=\left({\mathbf{I}}_{n}-\tilde{{\mathbf{D}}}\left(\tilde{{\mathbf{D}}}^{T}\tilde{{\mathbf{D}}}\right)^{-1}\tilde{{\mathbf{D}}}^{T}\right){\mathbf{X}}. $$

The standard PLS regression is then used on **X**_*Orth*_ instead of **X**. This avoids iterations in the algorithm since the residuals associated with $\tilde {{\mathbf {D}}}$ in the OLS of *Y* on $\left [\tilde {{\mathbf {D}}},{\mathbf {T}}\right ]$ are the same as the residuals of **Y** on $\tilde {{\mathbf {D}}}$ (the column space of $\tilde {{\mathbf {D}}}$ and the column space of **T** are orthogonal). Thus, the residuals do not change during the iterations avoiding the iterative process. This procedure is denoted by

$$\left({\mathbf{V}},\hat{{\boldsymbol{\gamma}}}^{\tilde{{\mathbf{D}}}},\hat{{\boldsymbol{\gamma}}}^{{\mathbf{X}}}\right) \longleftarrow \text{LS-PLS}({\mathbf{Y}},{{\mathbf{D}}},{\mathbf{X}},\kappa) $$

where **V** is the projection matrix, also called the loading matrix (of size *p*×*κ*), which allows us to compute **T** from **X** based on the relationship **T**=**XV**. The vector $\hat {{\boldsymbol {\gamma }}}^{\tilde {{\mathbf {D}}}}$ is the estimate of the vector, in which a coefficient exists for each column of $\tilde {{\mathbf {D}}}$. In the usual regression context, the loading matrix **V** allows us to compute the coefficients of $\hat {{\boldsymbol {\gamma }}}^{{\mathbf {X}}}$ using the coefficients in the dimension-reduced space $\hat {{\boldsymbol {\gamma }}}^{{\mathbf {T}}}$ with $\hat {{\boldsymbol {\gamma }}}^{{\mathbf {X}}}={\mathbf {V}} \hat {{\boldsymbol {\gamma }}}^{{\mathbf {T}}}$. In the LS-PLS context, when **X** is orthogonalized on $\tilde {{\mathbf {D}}}$, we can similarly compute the coefficient $\hat {{\boldsymbol {\gamma }}}^{{\mathbf {X}}}$, in which a coefficient exists for each column of **X**_*Orth*_ that is not of **X**. Note that for a new individual sample (**d**_0_^*T*^,**x**_0_^*T*^)^*T*^, the linear predictor associated with the LS-PLS methods is given by :

$$\hat{y}_{0}=\tilde{{\mathbf{d}}}_{0}^{T}\hat{{\boldsymbol{\gamma}}}^{\tilde{{\mathbf{D}}}}+ \left(x_{0}^{T}-\tilde{{\mathbf{d}}}_{0}^{T}\left(\tilde{{\mathbf{D}}}^{T}\tilde{{\mathbf{D}}}\right)^{-1}\tilde{{\mathbf{D}}}^{T}{\mathbf{X}}\right) \hat{{\boldsymbol{\gamma}}}^{{\mathbf{X}}}. $$

### Linear logistic regression - ridge penalty and RIRLS

For a typical designed experiment logistics model, let us consider a general design matrix **U** of size *n*×*m* and the response variable collected in a {0,1}^*n*^-valued vector **Y**. We denote **U**_*i*·_, the *i*-th row of **U**, and **Y**_*i*_ as the *i*-th element of **Y**. The conditional class probability, i.e., the conditional expectation of **Y**_*i*_ given **U**_*i*·_, defined by $\pi _{i}=\mathbb {P}({\mathbf {Y}}_{i}=1 \vert {\mathbf {U}}_{i\cdot }={\mathbf {u}}_{i})$, is related to the linear predictor ${\mathbf {\eta }}_{i} = \left [1 \; {\mathbf {u}}_{i}^{T}\right ] {\boldsymbol {\gamma }}$, with ${\boldsymbol {\gamma }} \in {\mathbb {R}}^{m+1}$ through the nonlinear relation *π*_*i*_=*h*(*η*_*i*_), where *h*(*η*_*i*_)=1/(1+ exp(−*η*_*i*_)). The parameter ***γ*** is unknown and must be estimated from the data. Vectors ***π*** and ***η*** depend on ***γ*** and should be written as ***π***^***γ***^ and ***η***^***γ***^, respectively. For the sake of clarity, we use only the notations ***π*** and ***η*** in this paper. In logistic discrimination, the estimation is usually carried out using $\hat {{\boldsymbol {\gamma }}}^{\text {ML}}$, i.e., the maximum likelihood (ML) estimator. The log-likelihood of the observations for the value ***γ*** of the parameter, simply denoted by *ℓ*(***γ***), is given by

$$\ell({\boldsymbol{\gamma}}) = \sum_{i=1}^{n} \left\{ y_{i} \eta_{i}- \ln \left(1 + \exp(\eta_{i}) \right)\right\}. $$

Let **W**(***γ***) be the diagonal *n*×*n* matrix with entries {**W**(***γ***)}_*ii*_=*π*_*i*_(1−*π*_*i*_). For a vector **u**_0_, the predicted class $\hat {{Y}_{0}}$ of the sample is given by $\hat {{Y}_{0}}=1_{(\hat {\pi }_{0}> 1-\hat {\pi }_{0})}$, where $\hat {\pi }_{0}=h\left (\left [1\; {\mathbf {u}}_{0}^{T}\right ]^{T} \hat {{\boldsymbol {\gamma }}}^{\text {ML}}\right) $ and 1_(·)_ is the indicator function. When this estimate exists, it is computed as the limit of a Newton-Raphson sequence; this algorithm is known as the iteratively reweighted LS algorithm [32], denoted by IRLS(**Y**,**U**). From step *t* to *t*+1, we have:

1 $$\begin{array}{@{}rcl@{}} {\mathbf{z}}^{(t)} &=& \tilde{{\mathbf{U}}} {\boldsymbol{\gamma}}^{(t)} + \left[ \mathbf{W}^{(t)} \right]^{-1} \left({\mathbf{Y}} - {\boldsymbol{\pi}}^{(t)} \right), \end{array} $$

2 $$\begin{array}{@{}rcl@{}}  {\boldsymbol{\gamma}}^{(t+1)} &=& \left(\tilde{{\mathbf{U}}}^{T} {\mathbf{W}}^{(t)} \tilde{{\mathbf{U}}} \right)^{-1} \tilde{{\mathbf{U}}}^{T} {\mathbf{W}}^{(t)} {\mathbf{z}}^{(t)}, \end{array} $$

where $\tilde {{\mathbf {U}}} {=} [1_{n} \; {\mathbf {U}}]$ and **W**^(*t*)^ is shorthand notation for **W**(***γ***^(*t*)^). The quantity ***π***^(*t*)^ is shorthand notation for the vector of size *n* whose *n*-th element is given by $h\left (\tilde {{\mathbf {U}}}_{i\cdot }^{T}{\boldsymbol {\gamma }}^{(t)}\right)$. The IRLS algorithm can thus be considered as an iteratively **W**(***γ***^(*t*)^)-weighted LS regression of a ${\mathbb {R}}^{n}$-valued pseudovariable **z**^(*t*)^ onto the columns of $\tilde {{\mathbf {U}}}$. Note that in some cases, including the practical case where *n*<<*m*, the existence and unicity of $\hat {{\boldsymbol {\gamma }}}^{\text {ML}}$ for logit models are not guaranteed. Thus, regularization methods such as the ridge penalty are required. The ridge estimator [33], denoted by $\hat {{\boldsymbol {\gamma }}}^{R}$, is defined as the (unique) maximizer of the penalized likelihood *ℓ*^∗^(***γ***)=*ℓ*(***γ***)−0.5*λ****γ***^*T*^***γ***, where *λ*>0 is the shrinkage parameter. We call this the Ridge-IRLS algorithm (RIRLS). It consists of replacing the weighted regression (2) in IRLS with a weighted ridge regression ${\boldsymbol {\gamma }}^{(t+1)} = \left (\tilde {{\mathbf {U}}}^{T} {\mathbf {W}}^{(t)} \tilde {{\mathbf {U}}}+ \lambda \tilde {{\mathbf {I}}}_{m+1} \right)^{-1} \tilde {{\mathbf {U}}}^{T} {\mathbf {W}}^{(t)} {\mathbf {z}}^{(t)}$, where **z**^(*t*)^ is built as in (1) and $\tilde {{\mathbf {I}}}_{m+1}$ is a diagonal matrix of size (*m*+1)×(*m*+1), the diagonal of which is equal to (0,1,…,1). We then define $\left (\hat {{\boldsymbol {\gamma }}}^{{\mathbf {U}}}, {\mathbf {z}}^{\infty },{\mathbf {W}}^{\infty }\right) \longleftarrow \text {RIRLS}({\mathbf {Y}},{\mathbf {U}},\lambda)$, where $\hat {{\boldsymbol {\gamma }}}^{{\mathbf {U}}}$ is the resulting estimator of ***γ***, and **z**^*∞*^ is the pseudoresponse variable (resp. the weight matrix **W**^*∞*^) at convergence of the algorithm. Note that when the model does not contain the intercept term (i.e., it uses **U** instead of $\tilde {{\mathbf {U}}}$), the matrix $\tilde {{\mathbf {I}}}_{m+1}$ is replaced with the identity matrix **I**_*m*_. The parameter *λ* controls the amount of shrinkage in the data and can be chosen from the data for instance, by a cross-validation procedure.

### Extensions of LS-PLS for logistic regression

Extending the LS-PLS approach to the framework of the logistic model is not straightforward. For instance, there are several ways to use PLS in the classification context. In the following section, we propose extending three of them [18–20] to LS-PLS for logistic regression.

#### Nguyen and Rocke’s approach.

To extend PLS to logistic regression, [18] first compute the score *n*×*κ* matrix **T** associated with the PLS regression of **Y** on **X**. Then, they estimate the parameter in the ML sense by running IRLS(**Y**,**T**). If we want to adapt this approach to LS-PLS, we have to replace the call to PLS step with LS-PLS(**Y**,**D**,**X**,*κ*) and then perform IRLS(**Y**,[**D** **T**]). The detailed procedure of this LS-PLS-IRLS method is as follows:

$${}\text{Step} 1. \left. \begin{array}{l} \left({\mathbf{V}},\hat{{\boldsymbol{\gamma}}}_{\text{aux}}^{\tilde{{\mathbf{D}}}}, \hat{{\boldsymbol{\gamma}}}_{\text{aux}}^{{\mathbf{X}}}\right) \longleftarrow \text{LS-PLS}({\mathbf{Y}},{{\mathbf{D}}},{\mathbf{X}},\kappa),\\[2ex] \end{array}\right. $$

$${}\text{Step} 2. \left| \begin{array}{l} {\mathbf{T}}={\mathbf{X}}{\mathbf{V}}, \\[2ex] \left(\left(\!\hat{{\boldsymbol{\gamma}}}_{\text{LS-PLS-IRLS}}^{\tilde{{\mathbf{D}}}},\hat{{\boldsymbol{\gamma}}}_{\text{aux}}^{{\mathbf{T}}}\right)\!,{\mathbf{z}}^{\infty}_{\text{aux}},{\mathbf{W}}^{\infty}_{\text{aux}}\right) \!\longleftarrow\! \text{RIRLS}({\mathbf{Y}},[{\mathbf{D}};{\mathbf{T}}],\lambda), \\[2ex] \hat{{\boldsymbol{\gamma}}}_{\text{LS-PLS-IRLS}}^{{\mathbf{X}}} \longleftarrow {\mathbf{V}}\hat{{\boldsymbol{\gamma}}}_{\text{aux}}^{{\mathbf{T}}}.\\[2ex] \end{array}\right. $$

Even if this method yields relatively good results in practice, note that applying PLS (in Step 1) with a binary input **Y** is unappealing. In addition, the PLS-regression step does not consider the heteroscedasticity of the response vector **Y**. The value of *κ* can be chosen by cross-validation.

#### Marx’s approach.

In [19], the authors introduce an algorithm that extends PLS to generalized linear models, so-called IRPLS. Specifically, IRPLS can be understood as an IRLS algorithm in which the weighted LS regression (2) is replaced with the PLS regression, PLS ([**W**^(*t*)^]^1/2^**z**^(*t*)^,[**W**^(*t*)^]^1/2^**X**,*κ*). Note that PLS applied with the maximal number of PLS components is the same as LS. Note that [19] chooses *κ*=rank(**X**); hence, when **X** is full row rank (which is often the case when *n*<<*p*), this algorithm never converges. Some authors (see, for instance, [34, 35]) use similar algorithms but with *κ*<rank(**X**). In this case, nothing ensures that this algorithm converges. As previously mentioned, if we want to adapt this approach for LS-PLS, we can simply replace the call to PLS with LS-PLS. This iterative process, called IR-LS-PLS, is detailed in the following algorithm.

Iterate until convergence,

$$\left| \begin{array}{l} \left({\mathbf{V}}^{(t+1)},\hat{{\boldsymbol{\gamma}}}^{\tilde{{\mathbf{D}}},(t+1)},\hat{{\boldsymbol{\gamma}}}^{{\mathbf{X}},(t+1)}\right)\longleftarrow\\ \text{LS-PLS}\left(\left[{\mathbf{W}}^{(t)}\right]^{1/2}{\mathbf{z}}^{(t)},\left[{\mathbf{W}}^{(t)}\right]^{1/2}{\mathbf{D}}, \left[{\mathbf{W}}^{(t)}\right]^{1/2}{\mathbf{X}},\kappa\right),\\ \text{update } {\mathbf{z}}^{(t)} \text{according to Eq. (1)} \text{ with} \tilde{{\mathbf{U}}}=\left[\tilde{{\mathbf{D}}},{\mathbf{X}}{\mathbf{V}}^{(t)}\right].\\ \end{array}\right. $$

$$\left. \begin{array}{l} \hat{{\boldsymbol{\gamma}}}_{\text{IR-PLS-IRLS}}^{\tilde{{\mathbf{D}}}}= \hat{{\boldsymbol{\gamma}}}_{\infty}^{\tilde{{\mathbf{D}}}},\\[2ex] \hat{{\boldsymbol{\gamma}}}_{\text{IR-PLS-IRLS}}^{{\mathbf{X}}} = \hat{{\boldsymbol{\gamma}}}_{\infty}^{{\mathbf{X}}},\\[2ex] \end{array} \right. $$

where [**W**^(*t*)^]^1/2^ is a square root matrix of **W**^(*t*)^ that satisfies [**W**^(*t*)^]^*T*/2^[**W**^(*t*)^]^1/2^=**I**_*n*_, $\hat {{\boldsymbol {\gamma }}}_{\infty }^{\tilde {{\mathbf {D}}}}$ and $\hat {{\boldsymbol {\gamma }}}_{\infty }^{{\mathbf {X}}}$ are coefficient estimates obtained at convergence. The drawback of this method is that convergence problems often occur. The parameter *κ* can also be selected by cross-validation.

#### Ridge partial least squares approach.

To extend PLS to the logistic regression model, [20] suggest replacing the binary data with a pseudoresponse variable whose expected value has a linear relationship with the covariates. The pseudoresponse variable **z**^*∞*^ at convergence of the RIRLS algorithm verifies this condition: it can be written as ${{\mathbf {z}}}^{\infty }={\mathbf {X}} \hat {{\boldsymbol {\gamma }}}^{R} +{{\boldsymbol {\varepsilon }}}$, where, $ \hat {{\boldsymbol {\gamma }}}^{R}$ subject to being the true value of the parameter, ***ε*** is a centered vector of covariance matrix (**W**^*∞*^)^−1^. This procedure is called R-PLS. As a consequence, in the same spirit, to extend LS-PLS to logistic regression, we can propose a procedure that combines the ridge penalty and LS-PLS, called R-LS-PLS. Let *λ* be some positive real constant and *κ* be some positive integer. R-LS-PLS is divided into two steps:

$${}\text{Step} 1. \left. \begin{array}{l} \left(\left(\hat{{\boldsymbol{\gamma}}}_{\text{aux}}^{\tilde{{\mathbf{D}}}}, \hat{{\boldsymbol{\gamma}}}_{\text{aux}}^{{\mathbf{X}}}\right),{{\mathbf{z}}}^{\infty},{\mathbf{W}}^{\infty}\right) \longleftarrow \text{RIRLS}({\mathbf{Y}},[{{\mathbf{D}}}\; {\mathbf{X}}],\lambda),\\[2ex] \end{array}\right. $$

$${}\text{Step} 2. \left. \begin{array}{r} \left(\!{\mathbf{V}},\hat{{\boldsymbol{\gamma}}}_{\text{R-LS-PLS}}^{\tilde{{\mathbf{D}}}},\hat{{\boldsymbol{\gamma}}}_{\text{R-LS-PLS}}^{{\mathbf{X}}}\!\right)\!\longleftarrow\! \text{LS-PLS}\left(\!\left[{\mathbf{W}}^{\infty}\right]^{1/2}{{\mathbf{z}}}^{\infty},\right.\\ \left.\left[{\mathbf{W}}^{\infty}\right]^{1/2}{{\mathbf{D}}},\left[{\mathbf{W}}^{\infty}\right]^{1/2}{\mathbf{X}},\kappa\right).\\[2ex] \end{array}\right. $$

The first step builds a continuous response variable **z**^*∞*^ for the input of LS-PLS, the “dispersion matrix” of which is [**W**^*∞*^]^−1^. This explains the weight [**W**^*∞*^]^1/2^ present in the second step. Note that in Step 1, we do not choose to regularize **D** with the ridge penalty. When the dimensions of matrix **X** are low, we may decide to not regularize it by putting *λ*=0 in Step 1. The R-LS-PLS method depends on two parameters, *λ* and *κ*, that can be selected by cross-validation.

These three approaches have been implemented in R software version 3.1.2, and an R package called lsplsGlm has been proposed to enhance their use.

## Additional files

Additional file 1 Supplement to the simulation study: Synthetic data with larger variances for influential variables. As reported in the simulation study of the paper, the noninfluential variables having the highest variance may seem unrealistic because the influential gene expression variables can have, in practice, higher variance than the noninfluential ones. We consider here the same example as in the simulation study but invert the variance levels. These simulation results are presented here. (PDF 130 kb)

Additional file 2 Supplement to the real data analysis: CNS data. Plots similar to those in Fig. 3 of the paper corresponding to *p*_*red*_ equal to 50, 100, and 750, respectively. (PDF 131 kb)

Additional file 3 Supplement to the real data analysis: breast cancer data. Plots similar to those in Fig. 5 of the paper corresponding to *p*_*red*_ equal to 50, 100, and 750, respectively. (PDF 129 kb)

Additional file 4 Supplement to the simulation study: Collinearity issue of the clinico-genomic integration. Results from simulation study addressed to evaluate the collinearity issue of the clinico-genomic integration are presented here. The simulation study is based on that presented in the paper. (PDF 177 kb)

## Abbreviations

AUC: Area under the curve
GLM: Generalized linear model
IRLS: Iteratively reweighted least squares algorithm
LS: Least- square
PCA: Principal component analysis
PCR: Principal component regression
SIS: Sure independence screening
