# Supplementary material for: Classification based on extensions of LS-PLS using logistic regression: application to clinical and multiple genomic data
Source: BMC Bioinformatics. 2018 Sep 6;19:314. doi: 10.1186/s12859-018-2311-2 (PMC6127926; doi:10.1186/s12859-018-2311-2)
Supplement: Supplementary file 1 — Supplement to the simulation study: Synthetic data with larger variances for influential variables. As reported in the simulation study of the paper, the noninfluential variables having the highest variance may seem unrealistic because the influential gene expression variables can have, in practice, higher variance than the noninfluential ones. We consider here the same example as in the simulation study but invert the variance levels. These simulation results are presented here. (PDF 130 kb) [file 12859_2018_2311_MOESM1_ESM.pdf]

Classification based on extensions of LS-PLS using logistic regression: application to clinical and multiple genomic data Additional File 1

C. Bazzoli* **and S. Lambert-Lacroix**†

**LJK - Université de Grenoble*
BP 53, 38041 Grenoble cedex 9, France
† Université de Grenoble / CNRS / UPMF / TIMC-IMAG
UMR 5525, Grenoble, F-38041, France
CAROLINE.BAZZOLI@UNIV-GRENOBLE-ALPES.FR
SOPHIE.LAMBERT-LACROIX@UNIV-GRENOBLE-ALPES.FR

Supplement to the simulation study: Synthetic data with larger variances for influential variables

As reported in the simulation study of the paper (Section 2), the noninfluential variables having the highest variance may seem unrealistic because the influential gene expression variables can have, in practice, higher variance than the noninfluential ones. We consider here the same example as in Section 2 but invert the variance levels. To accomplish this, we fix $c_1 = 1$, $c_2 = 2$, $c_3 = 4$, and $c_4 = 8$. The simulation results are summarized in Figure S1.1. We obtain similar results: the LS-PCR method leads to poorer performance even if κ_{max} is equal to 8.

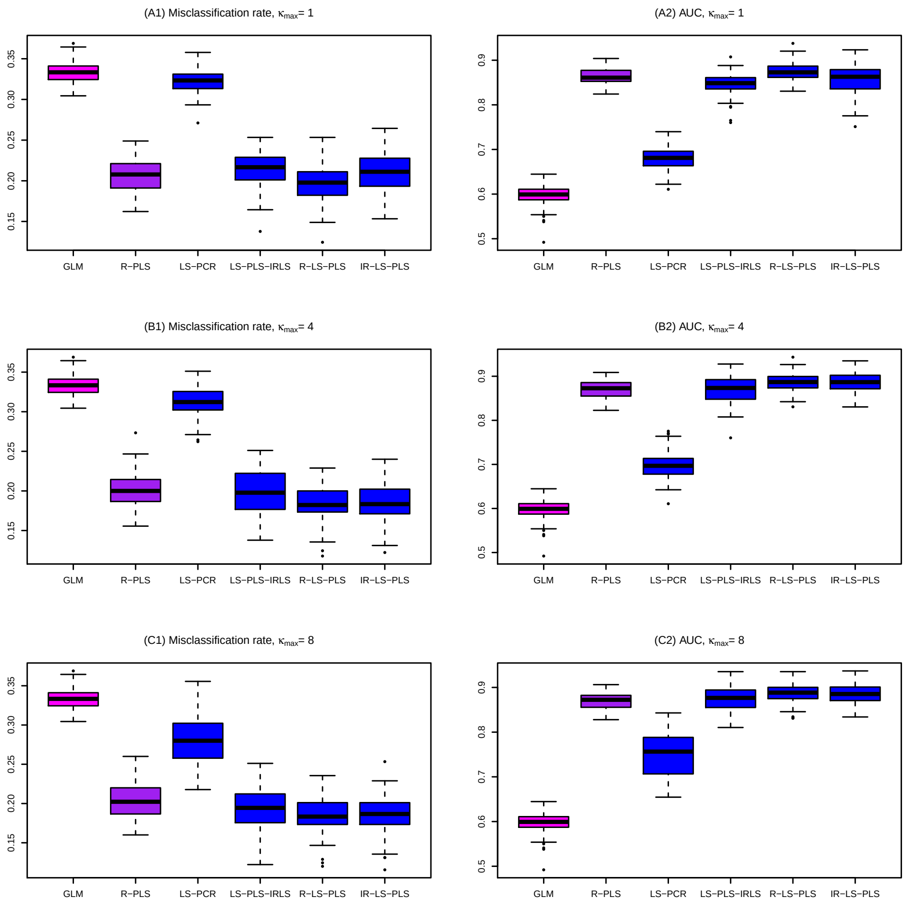

Figure S1.1: Boxplot of the misclassification rates (left part) and AUCs (right part) for the 100 simulated data sets. The results were obtained using the six methods and for different κ_{max}: (A1, A2): $\kappa_{max} = 1$; (B1, B2): $\kappa_{max} = 4$; (C1, C2): $\kappa_{max} = 8$. GLM and R-PLS denote the misclassification rates and AUCs obtained from applying the GLM to clinical data alone and PLS to gene expression data alone, respectively. LS-PCR denotes the approach derived from PCR, where gene expression data are analyzed using PCA and IRLS can thus be applied to the merged data set of PCA scores and clinical data. LS-PLS-IRLS, R-LS-PLS, and IR-LS-PLS denote the misclassification rates and AUCs obtained from the newly proposed LS-PLS approaches combining expression and clinical data. For clarity of the figure, we use a color code to indicate the predictions: pink when from clinical data alone, purple when from expression gene data alone and blue for the results of methods combining both types of variables. The number of gene expression variables to preselectp_{red} is set to 500 in the SIS procedure.
