# Supplementary material for: Classification based on extensions of LS-PLS using logistic regression: application to clinical and multiple genomic data
Source: BMC Bioinformatics. 2018 Sep 6;19:314. doi: 10.1186/s12859-018-2311-2 (PMC6127926; doi:10.1186/s12859-018-2311-2)
Supplement: Supplementary file 2 — Supplement to the real data analysis: CNS data. Plots similar to those in Fig. 3 of the paper corresponding to pred equal to 50, 100, and 750, respectively. (PDF 131 kb) [file 12859_2018_2311_MOESM2_ESM.pdf]

Classification based on extensions of LS-PLS using logistic regression: application to clinical and multiple genomic data
Additional File 2

C. Bazzoli* **and S. Lambert-Lacroix**†

LJK - Université de Grenoble
BP 53, 38041 Grenoble cedex 9, France
†*Université de Grenoble / CNRS / UPMF / TIMC-IMAG*
UMR 5525, Grenoble, F-38041, France
caroline.bazzoli@univ-grenoble-alpes.fr
sophie.lambert-lacroix@univ-grenoble-alpes.fr

Supplement to the real data analysis: CNS data

Figures S2.1, S2.2 and S2.3 show the results as in Figure 3 of the paper (Section 3.1) corresponding to p_{red} equal to 50, 100 and 750, respectively.

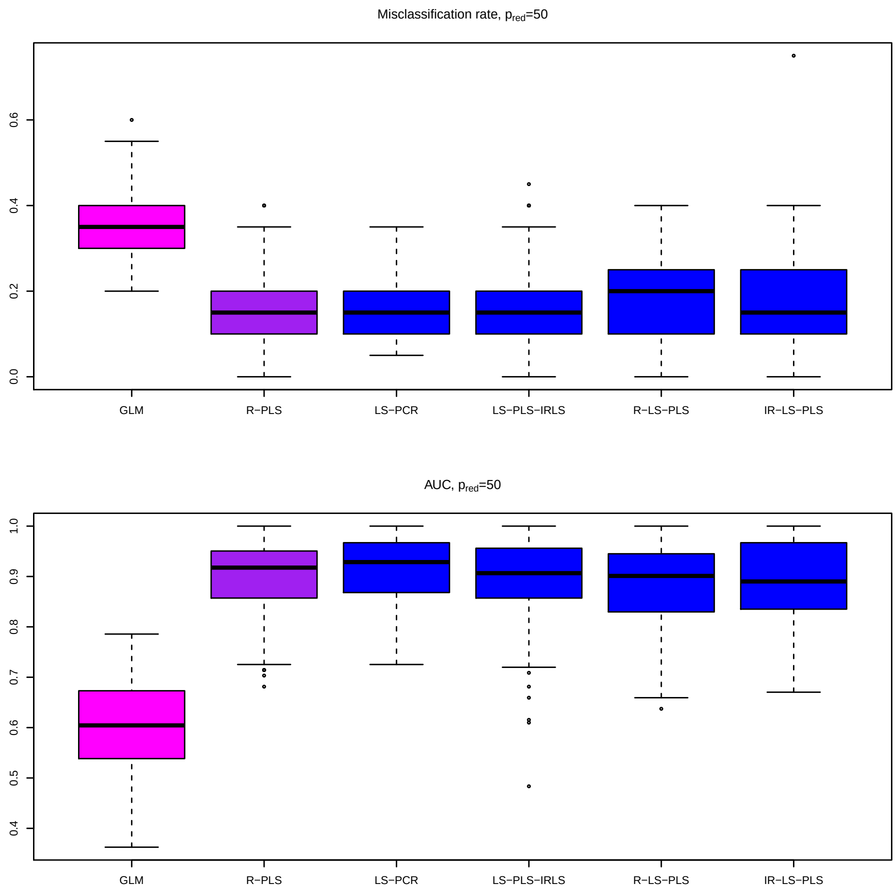

Figure S2.1: Boxplot of the misclassification rates (left part) and AUCs (right part) for CNS data estimated for 100 sampling using the six methods.The results were obtained using the six methods and for different κ_{max}: (A1, A2): $\kappa_{max} = 1$; (B1, B2): $\kappa_{max} = 4$; (C1, C2): $\kappa_{max} = 8$. GLM and R-PLS denote the misclassification rates and AUCs obtained from applying the GLM to clinical data alone and PLS to gene expression data alone, respectively. LS-PCR denotes the approach derived from PCR, where gene expression data are analyzed using PCA and IRLS can thus be applied to the merged data set of PCA scores and clinical data. LS-PLS-IRLS, R-LS-PLS, and IR-LS-PLS denote the misclassification rates and AUCs obtained from the newly proposed LS-PLS approaches combining expression and clinical data. For clarity of the figure, we use a color code to indicate the predictions: pink when from clinical data alone, purple when from expression gene data alone and blue for the results of methods combining both types of variables. The number of gene expression variables to preselect p_{red} is set to 50 in the SIS procedure.

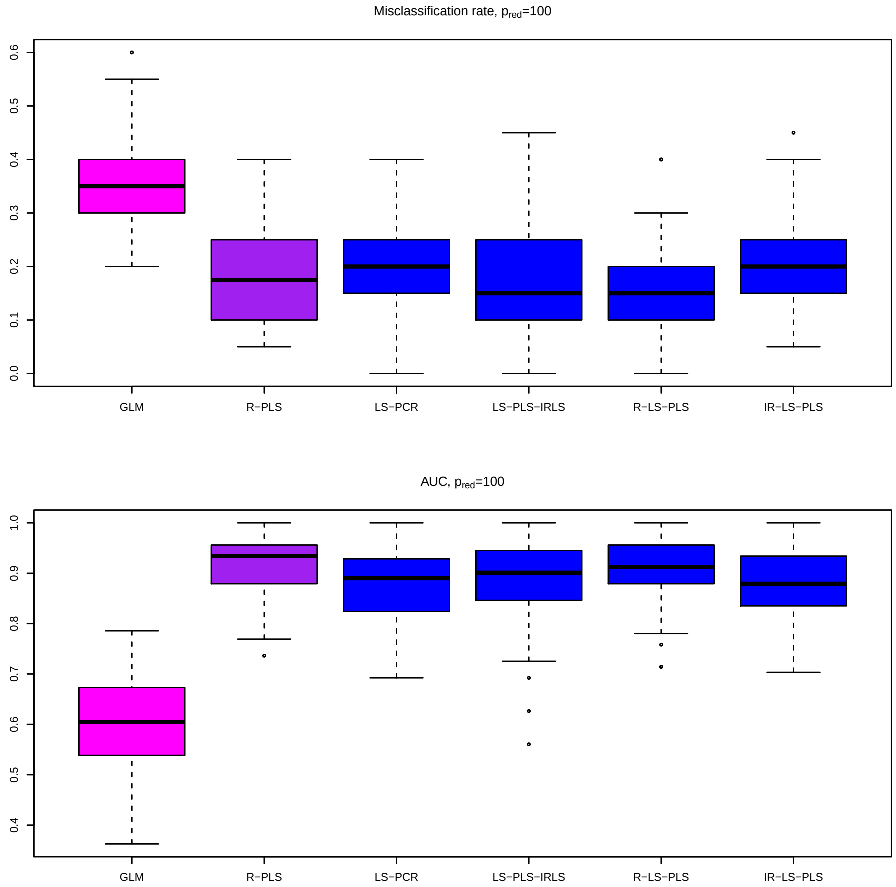

Figure S2.2: Boxplot of the misclassification rates (left part) and AUCs (right part) for CNS data estimated for 100 sampling using the six methods. The results were obtained using the six methods and for different κ_{max}: (A1, A2): $\kappa_{max} = 1$; (B1, B2): $\kappa_{max} = 4$; (C1, C2): $\kappa_{max} = 8$. GLM and R-PLS denote the misclassification rates and AUCs obtained from applying the GLM to clinical data alone and PLS to gene expression data alone, respectively. LS-PCR denotes the approach derived from PCR, where gene expression data are analyzed using PCA and IRLS can thus be applied to the merged data set of PCA scores and clinical data. LS-PLS-IRLS, R-LS-PLS, and IR-LS-PLS denote the misclassification rates and AUCs obtained from the newly proposed LS-PLS approaches combining expression and clinical data. For clarity of the figure, we use a color code to indicate the predictions: pink when from clinical data alone, purple when from expression gene data alone and blue for the results of methods combining both types of variables. The number of gene expression variables to preselect p_{red} is set to 100 in the SIS procedure.

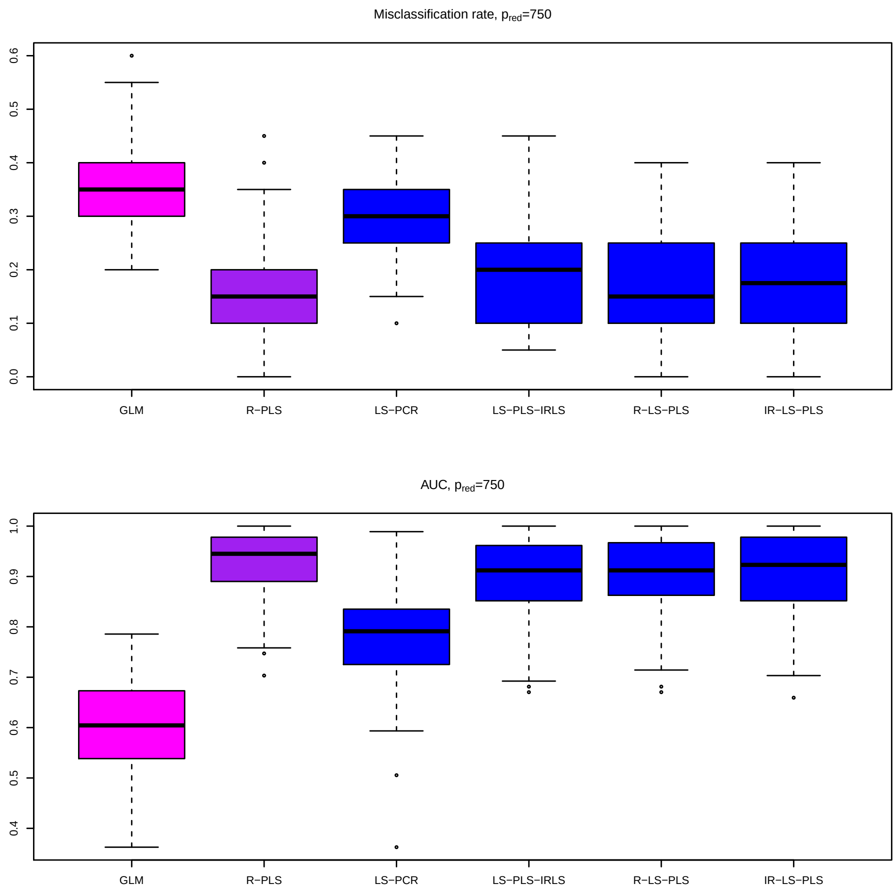

Figure S2.3: Boxplot of the misclassification rates (left part) and AUCs (right part) for CNS data estimated for 100 sampling using the six methods. The results were obtained using the six methods and for different κ_{max}: (A1, A2): $\kappa_{max} = 1$; (B1, B2): $\kappa_{max} = 4$; (C1, C2): $\kappa_{max} = 8$. GLM and R-PLS denote the misclassification rates and AUCs obtained from applying the GLM to clinical data alone and PLS to gene expression data alone, respectively. LS-PCR denotes the approach derived from PCR, where gene expression data are analyzed using PCA and IRLS can thus be applied to the merged data set of PCA scores and clinical data. LS-PLS-IRLS, R-LS-PLS, and IR-LS-PLS denote the misclassification rates and AUCs obtained from the newly proposed LS-PLS approaches combining expression and clinical data. For clarity of the figure, we use a color code to indicate the predictions: pink when from clinical data alone, purple when from expression gene data alone and blue for the results of methods combining both types of variables. The number of gene expression variables to preselect p_{red} is set to 750 in the SIS procedure.
