# Supplementary material for: Classification based on extensions of LS-PLS using logistic regression: application to clinical and multiple genomic data
Source: BMC Bioinformatics. 2018 Sep 6;19:314. doi: 10.1186/s12859-018-2311-2 (PMC6127926; doi:10.1186/s12859-018-2311-2)
Supplement: Supplementary file 3 — Supplement to the real data analysis: breast cancer data. Plots similar to those in Fig. 5 of the paper corresponding to pred equal to 50, 100, and 750, respectively. (PDF 129 kb) [file 12859_2018_2311_MOESM3_ESM.pdf]

Classification based on extensions of LS-PLS using logistic regression: application to clinical and multiple genomic data
Additional File 3

C. Bazzoli* **and S. Lambert-Lacroix**†

**LJK - Université de Grenoble*
BP 53, 38041 Grenoble cedex 9, France
†Université de Grenoble / CNRS / UPMF / TIMC-IMAG
UMR 5525, Grenoble, F-38041, France
CAROLINE.BAZZOLI@UNIV-GRENOBLE-ALPES.FR
SOPHIE.LAMBERT-LACROIX@UNIV-GRENOBLE-ALPES.FR

Supplement to the real data analysis: breast cancer data

Figures S3.1, S3.2 and S3.3 show the results as in Figure 5 of the manuscript (in Section 3.2) corresponding to p_{red} equal to 50, 100 and 750, respectively.

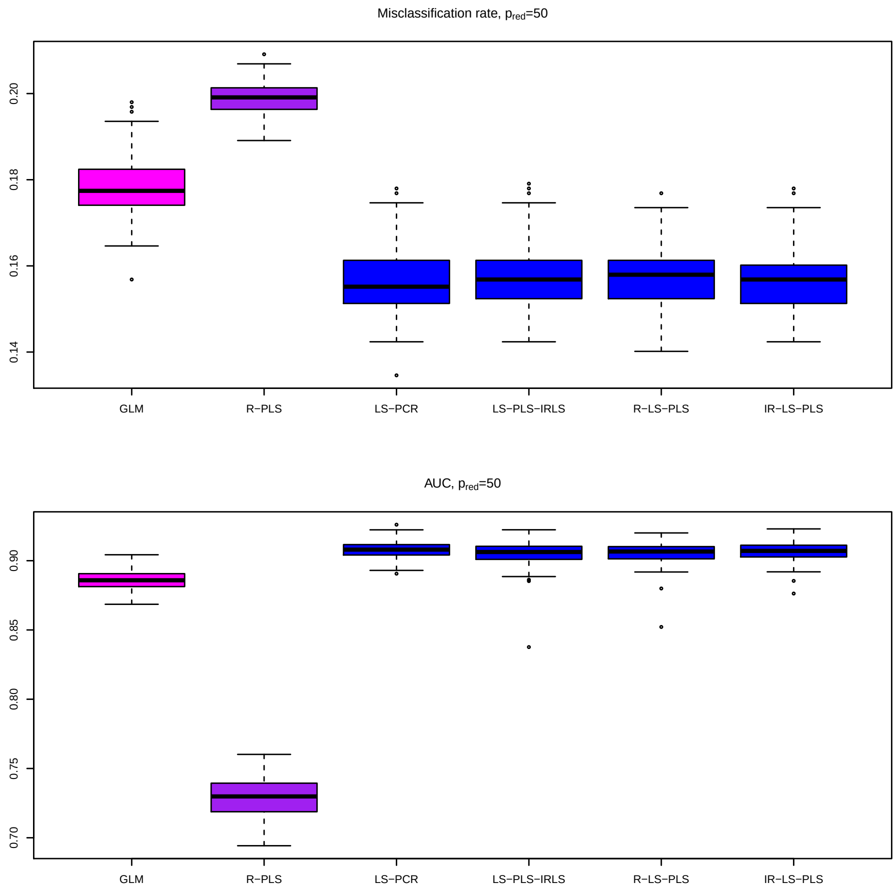

Figure S3.1: Distribution of misclassification rates and AUC for the somatic CNAs data estimated by 100 sampling using the six methods. The results were obtained using the six methods. GLM and R-PLS denote the misclassification rates and AUCs obtained from applying the GLM to clinical data alone and PLS to gene expression data alone, respectively. LS-PCR denotes the approach derived from PCR, where gene expression data are analyzed using PCA and IRLS can thus be applied to the merged data set of PCA scores and clinical data. LS-PLS-IRLS, R-LS-PLS, and IR-LS-PLS denote the misclassification rates and AUCs obtained from the newly proposed LS-PLS approaches combining expression and clinical data. For clarity of the figure, we use a color code to indicate the predictions: pink when from clinical data alone, purple when from expression gene data alone and blue for the results of methods combining both types of variables. The number of gene expression variables to preselect p_{red} is set to 50 in the SIS procedure.

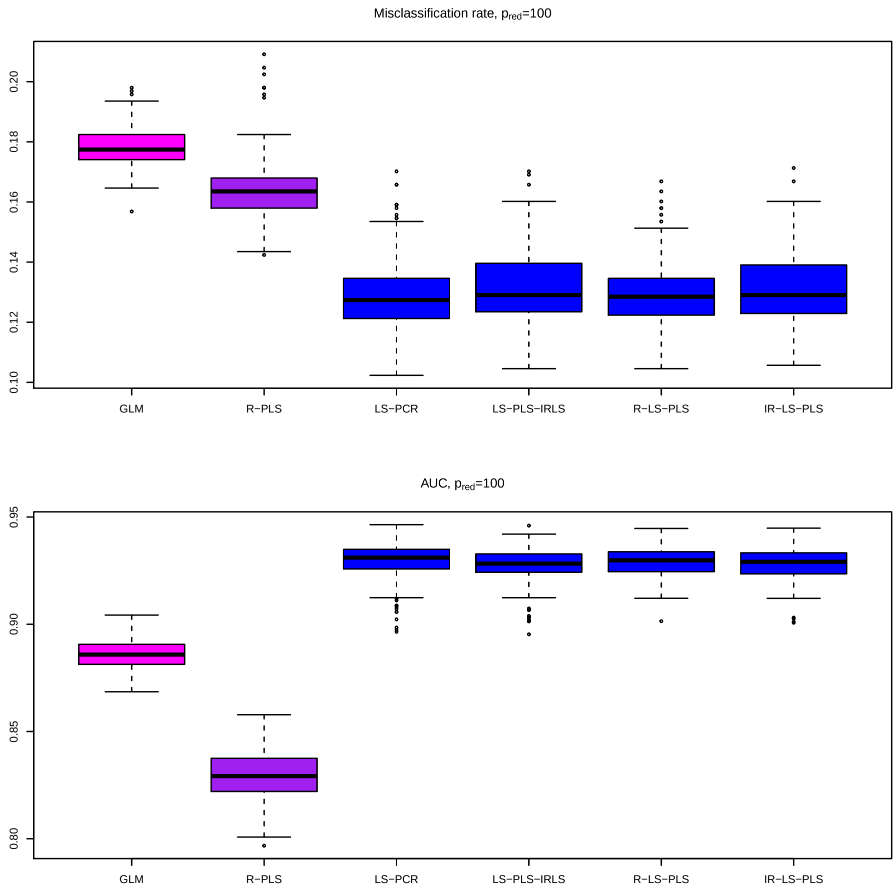

Figure S3.2: Distribution of misclassification rates and AUC for the somatic CNAs data estimated by 100 sampling using the six methods. The results were obtained using the six methods. GLM and R-PLS denote the misclassification rates and AUCs obtained from applying the GLM to clinical data alone and PLS to gene expression data alone, respectively. LS-PCR denotes the approach derived from PCR, where gene expression data are analyzed using PCA and IRLS can thus be applied to the merged data set of PCA scores and clinical data. LS-PLS-IRLS, R-LS-PLS, and IR-LS-PLS denote the misclassification rates and AUCs obtained from the newly proposed LS-PLS approaches combining expression and clinical data. For clarity of the figure, we use a color code to indicate the predictions: pink when from clinical data alone, purple when from expression gene data alone and blue for the results of methods combining both types of variables. The number of gene expression variables to preselect p_{red} is set to 100 in the SIS procedure.

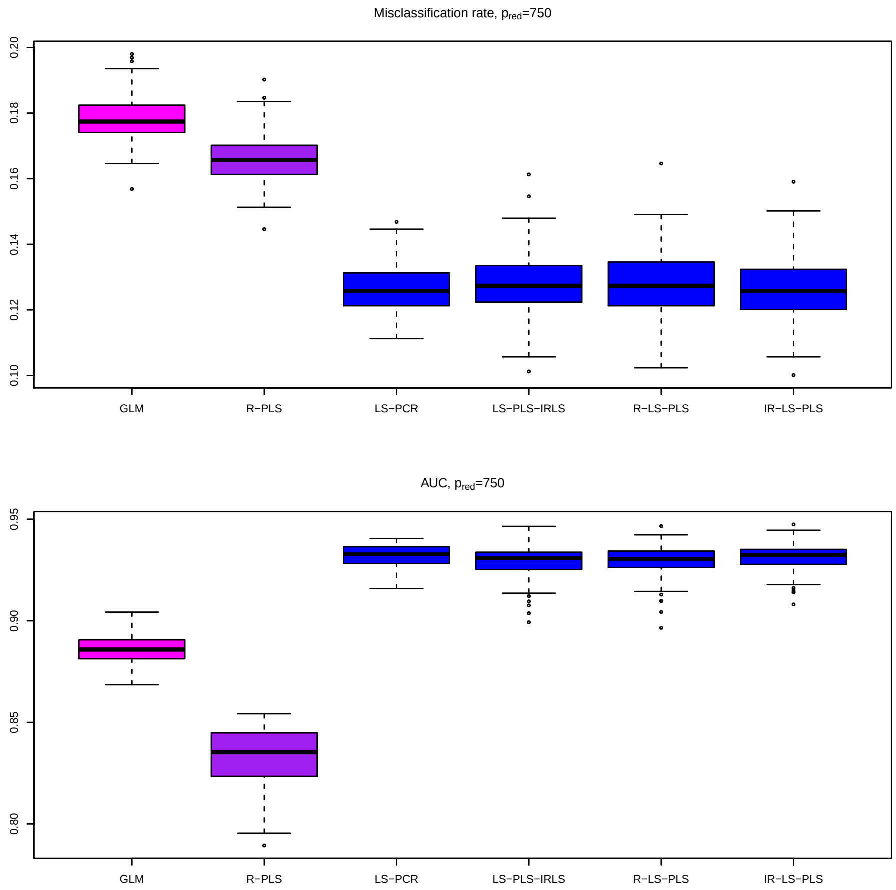

Figure S3.3: Distribution of misclassification rates and AUC for the somatic CNAs data estimated by 100 sampling using the six methods. The results were obtained using the six methods. GLM and R-PLS denote the misclassification rates and AUCs obtained from applying the GLM to clinical data alone and PLS to gene expression data alone, respectively. LS-PCR denotes the approach derived from PCR, where gene expression data are analyzed using PCA and IRLS can thus be applied to the merged data set of PCA scores and clinical data. LS-PLS-IRLS, R-LS-PLS, and IR-LS-PLS denote the misclassification rates and AUCs obtained from the newly proposed LS-PLS approaches combining expression and clinical data. For clarity of the figure, we use a color code to indicate the predictions: pink when from clinical data alone, purple when from expression gene data alone and blue for the results of methods combining both types of variables. The number of gene expression variables to preselect p_{red} is set to 750 in the SIS procedure.
