# Supplementary material for: Classification based on extensions of LS-PLS using logistic regression: application to clinical and multiple genomic data
Source: BMC Bioinformatics. 2018 Sep 6;19:314. doi: 10.1186/s12859-018-2311-2 (PMC6127926; doi:10.1186/s12859-018-2311-2)
Supplement: Supplementary file 4 — Supplement to the simulation study: Collinearity issue of the clinico-genomic integration. Results from simulation study addressed to evaluate the collinearity issue of the clinico-genomic integration are presented here. The simulation study is based on that presented in the paper. (PDF 177 kb) [file 12859_2018_2311_MOESM4_ESM.pdf]

# Classification based on extensions of LS-PLS using logistic regression: application to clinical and multiple genomic data Additional File 4

**C. Bazzoli*** **and S. Lambert-Lacroix**†

**LJK - Université de Grenoble*
*BP 53, 38041 Grenoble cedex 9, France*
*† Université de Grenoble / CNRS / UPMF / TIMC-IMAG*
*UMR 5525, Grenoble, F-38041, France*
CAROLINE.BAZZOLI@UNIV-GRENOBLE-ALPES.FR
SOPHIE.LAMBERT-LACROIX@UNIV-GRENOBLE-ALPES.FR

## Supplement to the simulation study: Collinearity issue of the clinico-genomic integration

The aim of this simulation study is to address the collinearity issue in the clinico-genomic integration. We simulated data sets, as in Section 2 of the paper, with the supplementary constraint that some variables of both matrices $\mathbf{D}$ and $\mathbf{X}$ are correlated. To do that, we first generated the matrix $\mathbf{X}$ of size $n \times p$ (with $p = 1000$), such as $\mathbf{X} = (\mathbf{X}^1, \mathbf{X}^2, \mathbf{X}^3, \mathbf{X}^4)$, where $\mathbf{X}^k \sim N(0_{bs^{(k)}}, \mathbf{\Sigma_X})$ with $\{\mathbf{\Sigma_X}\}_{ij} = \rho^{|i-j|}$ ,$k = 1, ...4$, $i, j = 1, ..., bs^{(k)}$ and, where $bs^{(1)} = bs^{(2)} = 475$, $bs^{(3)} = bs^{(4)} = 25$, and $\rho = 0.9$. Then, we generated the matrix $\mathbf{D}$ of size $n$ x $q$ (with $q = 4$). The columns $\mathbf{D}_{\cdot 1}$ and $\mathbf{D}_{\cdot 2}$ are independent and obtained using the following model :

$$\mathbf{D}_{\cdot k} = \mathbf{X}^3 \boldsymbol{\beta} + \epsilon_{\cdot k},\ k = 1, 2,$$

where $\boldsymbol{\beta}$ is one-vector of size 25 and $\epsilon_{jk}$, $j = 1, \ldots, n$, $k = 1, 2$, is a Gaussian residual term. The residual variance was simulated using three different values of signal-to-noise satio (SNR) equal to 1, 2 or 3, in order to consider different levels of collinearity of the clinico-genomic integration. We recall that higher is the SNR, stronger is the link between the variables $\mathbf{X}^3$ and $\mathbf{D}_{\cdot k}$. Based on the model described in the paper (Section 2), the columns $\mathbf{D}_{\cdot 3}$ and $\mathbf{D}_{\cdot 4}$ was generated using $N(0_2, \mathbf{\Sigma_D})$ with $\{\mathbf{\Sigma_D}\}_{ij} = \rho^{|i-j|}$, $i,\ j = 1, ..., 2$ and $\rho = 0.5$. We denote by $\mathbf{D}^{sc}$ the centred and scaled matrix $\mathbf{D}$. From these design matrices, we simulated $n = 100$ individuals with $Y_i \sim \mathcal{B}(\pi_i)$, where $\pi_i = [1\ \mathbf{D}^{sc}_{i\cdot}\ \mathbf{X}_{i\cdot}]\boldsymbol{\gamma}$ and $\boldsymbol{\gamma}$, the vector of estimated regression parameters, defined as, $\boldsymbol{\gamma} = [\gamma_1\ \boldsymbol{\gamma}_\mathbf{D}^T\ \boldsymbol{\gamma}_\mathbf{X}^T]^T$. We fixed $\gamma_1 = -2.5$, $\boldsymbol{\gamma}_\mathbf{D} = \{\{0.5\}^4\}$ and $\boldsymbol{\gamma}_\mathbf{X} = \{\{0\}^{475}, \{0\}^{475}, \{0.1\}^{25}, \{0.1\}^{25}\}$.

According to this model, we generated 100 training sets of size $n = 100$ and 100 test sets of size 450. We used a similar simulation framework as described in the Section 2 of the paper, with $\kappa_{max} = 4$ and $p_{red} = 500$.

The simulation results are summarized in Figure S4.1. Note that the results obtained using R-PLS are the same regardless of the $SNR$ value since the values of $\mathbf{X}$ are not affected. On the other hand, even if $\mathbf{D}$ matrices are impacted by the $SNR$, the results obtained by the GLM are quite stable. In this simulation study, regardless of the value of the SNR, the GLM approach, which uses only the clinical data, leads to better performance than R-PLS, which

uses only the genomic data. Concerning the other four methods, which combine expression and clinical data, R-LS-PLS returns the best results even compared to those of the GLM, regardless of the $SNR$ value. The two other extensions of LS-PLS yield results with more variability and perform poorly on average. The misclassification rates and AUCs improve once the $SNR$ increases, that is, collinearity increases. We believe that this outcome is due to the convergence issues observed for both LS-PLS extensions. Finally, the LS-PCR method is the one that evolves the most according to the $SNR$; the misclassification rates tend to decrease with higher levels of collinearity, though this approach remains less efficient than R-LS-PLS.

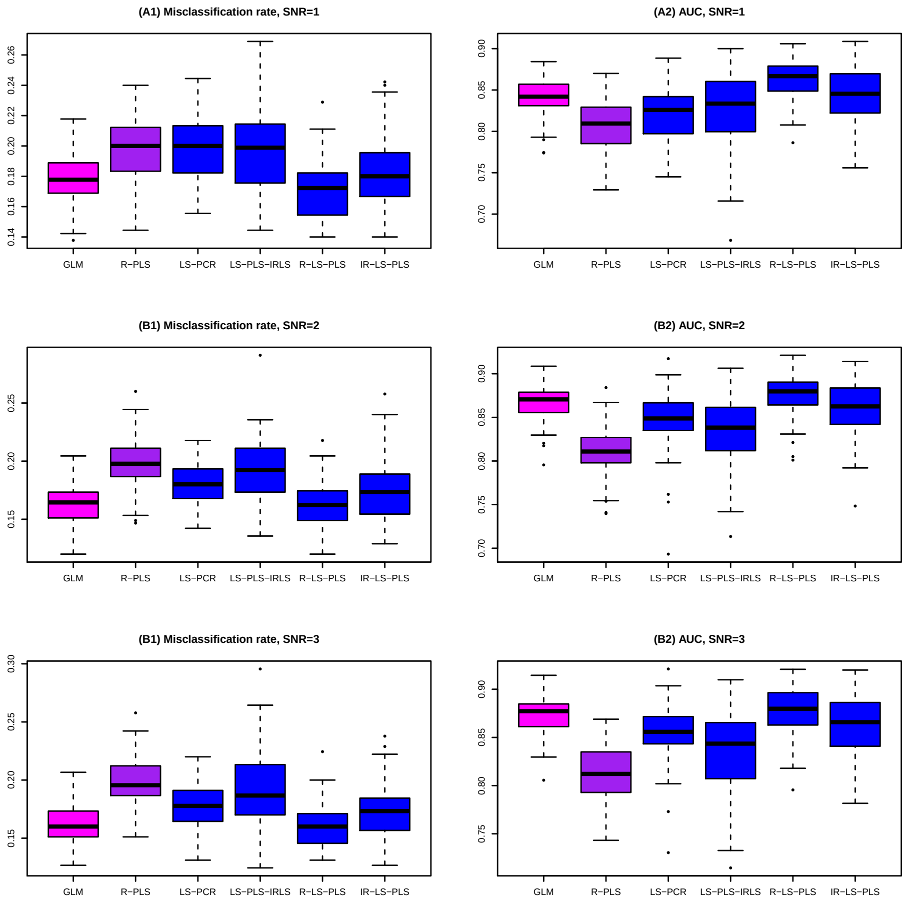


Figure S4.1: Boxplot of the misclassification rates (left part) and AUCs (right part) for the 100 simulated data sets. The results were obtained using the six methods and according to different $SNRs$ : (A1, A2): $SNR = 1$; (B1, B2): $SNR = 2$; (C1, C2): $SNR = 3$. GLM and R-PLS denote the misclassification rates and AUCs obtained from applying the GLM to clinical data alone and PLS to gene expression data alone, respectively. LS-PCR denotes the approach derived from PCR, where gene expression data are analyzed using PCA and IRLS can thus be applied to the merged data set of PCA scores and clinical data. LS-PLS-IRLS, R-LS-PLS, and IR-LS-PLS denote the misclassification rates and AUCs obtained from the newly proposed LS-PLS approaches combining expression and clinical data. For clarity of the figure, we use a color code to indicate the predictions: in pink when from clinical data alone, in purple when from expression gene data alone and blue for the results of methods combining both types of variables. The optimal number of PLS or PCR components is selected by choosing a value of $\kappa$ in the range from 1 to 4. From the SIS procedure, the relevant gene expression variables $p_{red}$ is set to 500. The boxplots are obtained without one error rate extreme value given by 0.41 and the corresponding AUC given by 0.68 for the IR-LS-PLS method.
